# Digital Care Pathway for Patients With Sleep Apnea in Specialized Care: Mixed Methods Study

**DOI:** 10.2196/47809

**Published:** 2024-02-22

**Authors:** Jari Haverinen, Terttu Harju, Hanna Mikkonen, Pia Liljamo, Miia Turpeinen, Jarmo Reponen

**Affiliations:** 1 Finnish Coordinating Center for Health Technology Assessment Oulu University Hospital Oulu Finland; 2 FinnTelemedicum, Research Unit of Health Sciences and Technology, Faculty of Medicine University of Oulu Oulu Finland; 3 Medical Research Center Oulu Oulu Pulmonary Department Oulu University Hospital and University of Oulu Oulu Finland; 4 The Wellbeing Services County of North Ostrobothnia Oulu Finland; 5 Finnish Institute for Health and Welfare Department of Knowledge Brokers Data and Analytics Unit Helsinki Finland; 6 Medical Research Center Oulu Oulu University Hospital and University of Oulu Oulu Finland

**Keywords:** health services, telehealth, telemedicine, health personnel, sleep apnea syndromes, mobile phone

## Abstract

**Background:**

Sleep apnea is a significant public health disorder in Finland, with a prevalence of 3.7%. Continuous positive airway pressure (CPAP) therapy is the first-line treatment for moderate or severe sleep apnea. From November 18, 2019, all patients who started their CPAP therapy at Oulu University Hospital were attached to a sleep apnea digital care pathway (SA-DCP) and were instructed on its use. Some patients still did not use the SA-DCP although they had started their CPAP therapy.

**Objective:**

We aimed to study health care professionals’ (HCPs’) perspectives on the SA-DCP and its usefulness for their work; whether the main targets of SA-DCP can be reached: shortening the initial guiding sessions of CPAP therapy, reducing patient calls and contact with HCPs, and improving patients’ adherence to CPAP therapy; and patients’ perspectives on the SA-DCP and its usefulness to them.

**Methods:**

Overall, 6 HCPs were interviewed in May and June 2021. The survey for SA-DCP users (58/91, 64%) and SA-DCP nonusers (33/91, 36%) was conducted in 2 phases: from May to August 2021 and January to June 2022. CPAP device remote monitoring data were collected from SA-DCP users (80/170, 47.1%) and SA-DCP nonusers (90/170, 52.9%) in May 2021. The registered phone call data were collected during 2019, 2020, and 2021. Feedback on the SA-DCP was collected from 446 patients between February and March 2022.

**Results:**

According to HCPs, introducing the SA-DCP had not yet significantly improved their workload and work practices, but it had brought more flexibility in some communication situations. A larger proportion of SA-DCP users familiarized themselves with prior information about CPAP therapy before the initial guiding session than nonusers (43/58, 74% vs 16/33, 49%; *P*=.02). Some patients still had not received prior information about CPAP therapy; therefore, most of the sessions were carried out according to their needs. According to the patient survey and remote monitoring data of CPAP devices, adherence to CPAP therapy was high for both SA-DCP users and nonusers. The number of patients’ phone calls to HCPs did not decrease during the study. SA-DCP users perceived their abilities to use information and communications technology to be better than nonusers (mean 4.2, SD 0.8 vs mean 3.2, SD 1.2; *P*<.001).

**Conclusions:**

According to this study, not all the goals set for the introduction of the SA-DCP have been achieved. Despite using the SA-DCP, some patients still wanted to communicate with HCPs by phone. The most significant factors explaining the nonuse of the SA-DCP were lower digital literacy and older age of the patients. In the future, more attention should be paid to these user groups when designing and introducing upcoming digital care pathways.

## Introduction

### Background

Sleep apnea is a significant public health disorder in Finland, with a prevalence of 3.7%. The prevalence of sleep apnea worldwide has been increasing in relation to the obesity pandemic [1]. Untreated sleep apnea increases cardiovascular diseases, accidents, likelihood of taking sick leave, and premature mortality [2]. The clinical severity of sleep apnea is defined based on 3 components: daytime sleepiness owing to sleep apnea, the apnea-hypopnea index (AHI), and arterial blood oxygen saturation [2]. Continuous positive airway pressure (CPAP) therapy is the first-line treatment for moderate or severe sleep apnea in addition to conservative therapy (ie, weight loss, avoidance of sleep-disturbing substances, and lifestyle issues) [2]. CPAP therapy is a safe and efficient treatment for sleep apnea, relieving both daytime and nighttime symptoms and improving traffic safety [3,4]. In Finland, the need for CPAP treatment and the number of outpatient visits in both specialized and primary care have increased considerably because of the increased number of patients with sleep apnea [5].

The digitalization of health care has been seen as a potential option for offering treatment to patients regardless of time and place and involving them in their own care [6,7]. In addition, digitalization has the potential to make health care systems more efficient [8]. Despite its potential to improve health care services, digitalization does not automatically guarantee better services [9]. It has been noted as a problem, for example, that digital services are not necessarily aligned with clinician and patient preferences [9]. The challenge is that, in some cases, they complement rather than substitute the current services, and care processes are not always redesigned to achieve the best benefits from digital services [9-12]. Citizens’ willingness and ability to use electronic services is also an obstacle to realizing the benefits of health care digitalization [13,14]. Because data breaches cause potentially catastrophic consequences, information security concerns have weakened patients’ adoption of digital health services [15,16]. The challenges of the technical implementation of digital services, such as missing functionalities and lack of interoperability with existing information systems, have weakened the willingness of health care professionals (HCPs) to use them [10].

Factors promoting the adoption of digital health services are their perceived benefits for patients and patients’ previous positive experiences with electronic services [13,15]. Previous studies showed that digital health interventions can improve patients’ adherence to their care [17,18]. For example, Aardoom et al [18] showed that adherence to CPAP therapy in patients with sleep apnea can be improved with digital interventions in the initial months of treatment. Adherence to the use of the digital health service has also been found in some studies to positively affect outcomes [19,20]. Good digital literacy promotes the use of digital health services; studies have found young people have better digital literacy than older age groups [13,21]. As the user base of digital health care services can be very broad, and users can have functional limitations owing to age or illness, the ease of use of these services is important in promoting their use [15,22].

Finland’s first phase of health care digitalization involved the digitalizing of HCPs’ tools, such as electronic patient records; e-prescribing and digitalization have progressed well [23]. Currently, Finnish citizens are increasingly offered digital health care services and products [22,24]. Several countries, including Finland, have introduced new health technology assessment methods to ensure that digital health provides evidence-based benefits [16,22,25]. Digital care pathways (DCPs) are an example of digital health care services, and today, there are >300 DCPs in use in Finnish specialized care units [26]. One of the main goals of DCPs is to complement or replace traditional health care appointment visits [26]. In addition, DCPs aim to support and help in the self-treatment of long-term illnesses, monitoring, and adaptation to the illness, as well as enable patients to prepare for various health care procedures beforehand [26]. Several DCPs have been studied in Finland from the perspective of HCPs, organizations, and patients [7,10,13,27-31]. One of these DCPs is the sleep apnea DCP (SA-DCP), which was introduced at Oulu University Hospital (OUH) on November 18, 2019 [32]. All patients who start their CPAP therapy in OUH will be attached to the SA-DCP, that is, their patient data will be recorded in it, and they will be instructed on how to log in and use it [32]. When a patient starts on the SA-DCP, they register as an SA-DCP user through strong identification by accepting the terms of use and privacy statement and entering his or her contact information [33].

### Objectives

In OUH, the CPAP therapy for patients with sleep apnea begins with an initial guiding session where patients are instructed on using their CPAP device. SA-DCP contains information and instructions about CPAP therapy; therefore, it would be desirable for patients to familiarize themselves with that information in advance [32]. In this way, the initial guiding session of CPAP therapy could be shortened because the basic information about CPAP therapy would not need to be reviewed again during the sessions. The SA-DCP contains reliable information about sleep apnea, its treatment, and CPAP therapy [32]. With the introduction of SA-DCP, it would be desirable to reduce patients’ phone calls and other contacts with HCPs when information can be found in the SA-DCP. The SA-DCP also includes electronic messaging between patients and HCPs, which could reduce such calls [32]. The major aim of the SA-DCP is to increase patients’ adherence to CPAP therapy. However, there is still a challenge in that some patients with sleep apnea do not log in and use it.

The main aims of the study are as follows:

1. To investigate HCPs’ perspectives on the SA-DCP and its usefulness for their work
2. To determine whether the main targets of SA-DCP can be reached: shortening the initial guiding sessions of CPAP therapy, reducing patient calls and contact with HCP, and increasing patients’ adherence to CPAP therapy.
3. To examine patients’ perspectives on the SA-DCP and its usefulness.

## Methods

### Study Participants and Data Collection

The study population included HCPs at the OUH and patients who had started their CPAP therapy at the OUH. The patient population consisted of 2 groups. *SA-DCP users* were patients who had registered with the SA-DCP. *SA-DCP nonusers* referred to patients who had not registered with the SA-DCP.

### Interviews of HCPs

HCPs of the OUH were contacted via email. Overall, 6 HCPs participated in the interviews from May to June 2021. Of these, 4 (67%) HCPs worked with patients, 1 (17%) was a supervisor, and 1 (17%) connected patients with sleep apnea to the SA-DCP and booked their appointments. The interviews were conducted remotely using a structured questionnaire. The HCPs provided voluntary informed consent for the interview by submitting a signed document. The interviews were then recorded and transcribed.

### Survey for Patients With Sleep Apnea

The first part of material collection was conducted between May and August 2021. With the help of OUH HCPs, the survey, along with an invitation to participate and information about it, was sent to SA-DCP nonusers by mail. Respondents could send their responses by prepaid mail or electronically using Webropol Ltd’s Webropol survey tool. SA-DPC users were informed about the study through the SA-DCP. They provided their consent and answered the survey using the SA-DCP questionnaire.

The second part of material collection was conducted between January and June 2022. Both SA-DCP users and SA-DCP nonusers were informed about the study with the annual device delivery in an assistive equipment center (AEC). They could send their responses by prepaid mail or answer electronically using Webropol Ltd’s Webropol survey tool.

The patients’ survey included multiple choice questions, 5-item Likert-type questions (with choices ranging from strongly disagree to strongly agree), and open-ended questions. In total, 33 SA-DCP nonusers and 58 SA-DCP users responded to the survey.

### Remote Monitoring Data of CPAP Devices

Information about patients’ adherence to CPAP therapy was collected from the remote monitoring data of CPAP devices. The HCP of OUH carried out the material collection manually in May 2021 in connection with 1-year controls of CPAP therapy. The collected information was anonymized and provided to the researchers. In total, CPAP remote monitoring data were collected from 90 SA-DCP nonusers and 80 SA-DCP users.

### Registered Data of Phone Calls

The information about the number of patients’ phone calls per year to an AEC was collected from Aurora Innovation Ltd’s TeleQ program. The registered phone call data were collected during 2019, 2020, and 2021.

### SA-DCP Customer Feedback Survey

Patients using the SA-DCP had the opportunity to provide customer feedback using the SA-DCP survey tool. The patients provided informed consent through the SA-DCP that their customer feedback could also be used for research purposes. The customer feedback did not contain any personal information. Feedback on the SA-DCP from 446 patients between February 18 and March 24, 2022, was included in this study.

### Statistical Methods

Patients’ survey data were analyzed using SPSS software (version 28.0; IBM Corp). Descriptive statistics were applied to calculate the mean and SD for continuous data and frequency and percentage for categorical data. Baseline differences between the groups were explored using a 2-tailed independent sample *t* test for continuous variables and chi-square test for categorical variables. A *P* value <.05 was considered statistically significant for all analyses.

### Qualitative Analysis

Qualitative methods were used in this study to analyze the open-ended questions in patient surveys and interviews with HCPs. The collected material was first analyzed using an inductive content analysis method to obtain a comprehensive understanding [34]. Initially, the HCPs’ and patients’ responses to the open-ended questions were open coded. Subsequently, the analyzed data were grouped into subcategories, and then similar findings were combined into the main categories to enable the final analysis. Finally, the textual data were analyzed using the quantification method [35].

### Ethical Considerations

The study followed the guidelines of the Finnish Advisory Board on Research Integrity [36]. According to Finnish Law (488/1999), this study was exempted from review by the institutional review board (ethics committee of Northern Ostrobothnia Hospital District). The respondents were informed of the study. All participants voluntarily participated in the study and provided their informed consent. The results were processed such that no participants were identifiable in the results or quotations of this study. Sensitive personal information was not collected. The data were processed and stored in a secure environment according to the procedures of the University of Oulu.

## Results

### The Number of New CPAP Therapies, SA-DCP Users, and Phone Calls in the Years Studied

The number of new CPAP therapies in OUH between 2019 and 2021 is presented in Table 1. The percentage of SA-DCP users has increased annually, but there are still patients who do not use the SA-DCP (Table 1). The number of phone calls per year to an AEC is presented in Table 1.

**Table 1:** New continuous positive airway pressure (CPAP) therapies, patients attached to the sleep apnea digital care pathway (SA-DCP), phone calls to an assistive equipment center (AEC) per year, and percentage of SA-DCP users.

	Year		
	2019	2020	2021
The number of new CPAP therapies	1172	1645	1160
The number of patients attached to the SA-DCP	292^a^	1645	1160
The number of SA-DCP users	130	1006	935
Percentage of SA-DCP users	44.5	61.2	80.6
The number of phone calls per year to an AEC	2784	4068	4020

^a^The SA-DCP was introduced from November 18, 2019, onward.

### HCPs’ Perspectives on the SA-DCP and its Usefulness for Their Work

On the basis of the interviews with HCPs, the main themes, facilitators, and barriers related to using the SA-DCP are presented in Textbox 1. According to the interviewed HCPs, they were unable to identify significant changes in their workload and working practices following the introduction of the SA-DCP. Only one responder perceived that his workload had slightly increased because the SA-DCP did not support integration with electronic patient record; therefore, patient data had to be transferred manually from one program to another (Textbox 1). However, HCPs reported that in some situations, the SA-DCP brought more flexibility to their work practices regarding patient communication (Textbox 1). For example, it enabled them to respond to patients’ DCP messages during nonurgent work times, not only prereserved times. HCPs also reported that the initial guiding session of CPAP therapy went more smoothly for SA-DCP users who had familiarized themselves with the information about CPAP therapy through the SA-DCP (Textbox 1). The interviewed HCPs hoped that patients would make more use of the SA-DCP and its possibilities so its benefits would be better used.

**Themes and perceived barriers and facilitators regarding implementation of the sleep apnea digital care pathway (SA-DCP) according to health care professionals (HCPs).**

**Use rate of SA-DCP**

- Barriers
  SA-DCP’s use rate had been lower than HCPs assumed it would be.
  Some patients still thought that the only proper contact was personal contact with HCPs.
  Patients’ previous experiences with the need to log in to several digital health care services reduced their motivation to use them.
- Facilitators
  Reminder text messages about logging into the SA-DCP have been sent to patients since June 2020.

**Initial guiding session of continuous positive airway pressure (CPAP) therapy**

- Barriers
  Some SA-DCP users and nonusers still had not familiarized themselves with the prior information about CPAP therapy in advance.
- Facilitators
  The guidance went more smoothly for SA-DCP users who familiarized themselves with the prior information about CPAP therapy.
  From the patients’ perspective, the instructional videos available in the SA-DCP were perceived as useful and clear.

**Patients’ communication practices with HCP**

- Barriers
  There were still a lot of phone calls.
  HCPs also had to be reminded that they should not always call patients in connection with treatment controls but send a message via the SA-DCP.
- Facilitators
  The SA-DCP gives patients more flexibility to contact HCPs regardless of time and place. For example, the patient may be in a location where they cannot answer the HCP’s phone call.
  HCPs may instruct the patient during a phone call to watch SA-DCP’s educational video to get a better understanding of the matter.

**Patients’ adherence to CPAP therapy**

- Barriers
  There was no clear indication that patients’ adherence to CPAP therapy was higher with the introduction of the SA-DCP.
- Facilitators
  Reports obtained from CPAP devices had increased some patients’ adherence to CPAP therapy.

**Integration of SA-DCP into existing information and communications technology systems**

- Barriers
  The SA-DCP had to be used in a different web browser than electronic patient record (EPR).
  Remote monitoring of CPAP devices requires a separate program, and remote monitoring data cannot be viewed via the SA-DCP.
  Attaching patients to the SA-DCP is laborious and must be done manually by copying patient information from the EPR.
  Data had to be copied manually from SA-DCP’s messages into patients’ care plans.

**Workload and work practices of HCPs**

- Barriers
  HCPs’ workloads did not change with the introduction of the SA-DCP.
- Facilitators
  The SA-DCP brought more flexibility to HCPs’ work practices regarding communication with patients.
  The SA-DCP was a good way to deliver the necessary contact information to patients and thereby instruct them to reserve time for the necessary procedures by themselves.

The professionals also brought up ideas for the development of SA-DCP. They hoped that SA-DCP’s integration with other information and communications technology (ICT) systems would be improved. One factor that caused a large workload for professionals was arranging appointment times for patients. The time reserved for the patient may not always suit him or her, necessitating a discussion about a more suitable time. If the patient could book appointments through the SA-DCP, it would greatly reduce the professionals’ working hours. Two respondents mentioned that in the future, an initial guiding session of CPAP therapy could also be carried out remotely, but this would require that patients for whom this would be suitable should be identified in advance. The professionals hoped that all surveys and measurements made by the patients related to their treatment would be available in an electronic format. The hope was also that the SA-DCP’s calendar would automatically remind patients, for example, to renew equipment, giving them more responsibility for managing their own affairs. One respondent wished that instructional videos could be directly linked to SA-DCP’s messages so that patients would not have to search for them.

The HCP interviewees perceived digital services in health care as a positive thing. According to them, the services should be easy to use, and the real end users of the services should be included in their development. One respondent believed that patients will use digital health care services more frequently in the future, but such systems are always initially met with resistance. The respondent mentioned that at first, patients in Finland were against e-prescribing and the Patient Data Repository of Kanta Services, but today, such services are commonplace, and people use them smoothly.

### Comparison of Characteristics Between SA-DCP Users and SA-DCP Nonusers

According to the patients’ survey, there were no statistically significant differences in age, sex, and smoking status between SA-DCP users and nonusers (Table 2). According to the remote monitoring data of CPAP devices, SA-DCP nonusers were older than SA-DCP users (mean 59.1, SD 13.8 vs mean 55.3, SD 10.8; *P*<.049; Table 3). Compared with nonusers, SA-DCP users perceived their own abilities to use ICT to be better (mean 4.2, SD 0.8 vs mean 3.2, SD 1.2; *P*<.001); they used computers, tablets, or smartphones more often (58/58, 100% vs 27/33 81%; overall *P*=.002); and they were more accustomed to using electronic services (mean 4.8, SD 0.5 vs mean 4.1, SD 1.2; *P*=.006; Table 2). There was no statistically significant difference in how regularly SA-DCP users and nonusers used the electronic services (Table 2). SA-DCP users thought that communication about SA-DCP and how to log in had been clear, although SA-DCP nonusers thought that it had not (yes 52/58, 91% vs yes 7/33, 24%; overall *P*<.001; Table 2). Compared with SA-DCP users, SA-DCP nonusers preferred phone calls or physical appointments with HCPs to manage their health-related issues (Table 2). Neither SA-DCP users nor SA-DCP nonusers had any major concerns about the data security and protection of digital health care services (Table 2).

**Table 2:** Patient responses to the survey.

Characteristics			Use of SA-DCP^a^					*P* value
			Nonusers (n=33)		Users (n=58)			
Age (years), mean (SD)			61.9 (11.6)		57.3 (12.0)		.08	
**Sex, n (%)**								.07
	Male	26 (79)		34 (59)				
	Female	7 (21)		24 (41)				
**Physical training frequency, n (%)**								.03
	Daily	16 (49)		15 (26)				
	Weekly	13 (39)		37 (64)				
	Monthly	1 (3)		5 (9)				
	Less than monthly	1 (3)		0 (0)				
	No physical training	2 (3)		1 (2)				
**Smoking,** **n (%)**								>.99
	Yes	2 (6)		4 (7)				
	No	31 (94)		53 (93)				
Adherence to CPAP^b^ therapy (own assessment; Likert scale 1-5), mean (SD)			4.8 (0.5)		4.7 (0.8)		.87	
**Patients familiar with CPAP therapy before the initial guiding session**			16 (49)		43 (74)		.02	
	The patient became familiar through SA-DCP, n (%)	N/A^c^		29 (67)		—^d^		
Average use of the CPAP device per night (hours), mean (SD)			6.3 (1.0)		6.3 (1.3)		.97	
**Has CPAP therapy helped the patient’s sleep apnea?,** **n (%)**								>.99
	Yes	28 (85)		49 (85)				
	No	0 (0)		0 (0)				
	Cannot say	5 (15)		9 (15)				
Information and communication technology skills (own assessment; Likert scale 1-5), mean (SD)			3.2 (1.2)		4.2 (0.8)		<.001	
**Patient’s computer, tablet, or smartphone use,** **n (%)**								.002
	Regularly (weekly)	27 (82)		58 (100)				
	Randomly (less often than weekly)	4 (12)		0 (0)				
	None	2 (6)		0 (0)				
How accustomed is the patient to using electronic services (eg, banking services, appointment services, etc; own assessment)? (Likert scale 1-5), mean (SD)			4.1 (1.2)		4.8 (0.5)		.006	
**If the patient uses electronic services, how regularly?, n (%)**								.55
	Daily	20 (69)		44 (76)				
	Weekly	7 (24)		11 (19)				
	Monthly	1 (3)		3 (5)				
	Less often than monthly	1 (3)		0 (0)				
**Would the patient choose an electronic service or a phone call as a contact method regarding her or his treatment?, n (%)**								<.001
	Electronic service	13 (39)		48 (83)				
	Phone call	20 (61)		10 (17)				
**If the patient could choose either an electronic service (eg, remote consultation) or a physical appointment regarding her or his treatment, which method would she or he prefer?, n (%)**								.048
	Electronic service	10 (30)		31 (53)				
	Physical appointment	23 (70)		27 (46)				
Patient concerns about the data security and protection of digital health care services (Likert scale 1-5), mean (SD)			2.6 (1.2)		2.2 (1.1)		.23	
**Has communication about SA-DCP and how to log in to it been sufficiently clear?, n (%)**								<.001
	Yes	7 (24)		52 (91)				
	No	22 (76)		5 (9)				
**Did SA-DCP increase the patient’s adherence to CPAP therapy?, n (%)**								
	Yes	N/A		42 (72)				
	No	N/A		16 (28)				
**Did the patient contact HCP^e^ during her or his treatment period?, n (%)**			17 (52)		33 (57)		.67	
	Through SA-DCP	N/A		17 (52)				
	Through another contact method	17 (100)		16 (48)				
**The contact was related to (total), n**			26		37		—	
	Treatment of sleep apnea, n (%)	8 (31)		3 (8)		—		
	CPAP therapy, n (%)	15 (58)		23 (62)		—		
	Other issues, n (%)	3 (12)		11 (30)		—		
**Did patients who contacted HCP get the help they needed?, n (%)**								
	Yes	16 (94)		31 (94)		.41		
	Through SA-DCP messaging	N/A		16 (52)		—		
	Through another contact method	16 (100)		15 (48)		—		
**Did the patient need to find additional information about his or her treatment without contacting HCPs during the treatment period?, n (%)**			9 (27)		23 (40)		.48	
	The patient got the information she or he needed	8 (89)		21 (91)		>.99		
	Through SA-DCP	N/A		8 (38)		—		
	Through another source (internet, patient organizations, etc)	8 (100)		13 (62)		—		

^a^SA-DCP: sleep apnea digital care pathway.

^b^CPAP: continuous positive airway pressure.

^c^N/A: not applicable.

^d^Not available.

^e^HCP: health care professional.

**Table 3:** Remote monitoring data of continuous positive airway pressure (CPAP) devices.

Characteristics			Use of SA-DCP^a^				*P* value
			Nonusers (n=90)		Users (n=80)		
Age (years), mean (SD)			59.1 (13.8)		55.3 (10.8)		.049
**Sex, n (%)**							.35
	Male	48 (53.3)		49 (61.3)			
	Female	42 (46.7)		31 (38.8)			
AHI^b^ at diagnosis, mean (SD)			32.5 (18.0)		30.6 (18.9)		.51
AHI residual in treatment, mean (SD)			2.4 (2.7)		2.1 (3.9)		.51
Percentage of nights CPAP was used, mean (SD)			92.6 (12.7)		91.5 (18.8)		.66
Hours of CPAP use per night, mean (SD)			6.2 (1.5)		6.1 (1.8)		.69
CPAP device mask leak, mean (SD)			3.1 (6.1)		2.8 (3.2)		.74
CPAP device median pressure, mean (SD)			8.4 (2.2)		7.8 (2.0)		.06

^a^SA-DCP: sleep apnea digital care pathway.

^b^AHI: apnea-hypopnea index.

### Patients’ Rationales for Using or Not Using SA-DCP

Patients were asked about their rationale for using or not using the SA-DCP (Table 4). SA-DCP users mostly adopted the SA-DCP because they thought that signing up for the SA-DCP was part of their treatment process (42/58, 72%). SA-DCP nonusers did not adopt the SA-DCP mainly because they were unaware of it (15/33, 46%).

**Table 4:** Patients’ rationales for using or not using the sleep apnea digital care pathway (SA-DCP).

Patients’ rationales for using or not using the sleep apnea digital care pathway (SA-DCP)			Values, n (%)
**Patients’ rationales for using the SA-DCP** **(n=58)**			
	I thought signing up for SA-DCP was part of my treatment process	42 (72)	
	It was recommended to me	39 (67)	
	It allows me to take care of my affairs regardless of time and place	31 (53)	
	I am very accustomed to using electronic services	25 (42)	
	I can more easily get information about sleep apnea and its treatment	18 (31)	
	I prefer to use electronic services for my treatment	15 (26)	
	I can more easily get information about CPAP^a^ therapy	15 (26)	
	I can take care of things related to my care more safely during the current COVID-19 period	12 (21)	
	By using SA-DCP, I am more committed to my treatment	8 (14)	
	Other reasons	2 (3)	
**Patients’ rationales for not using the SA-DCP** **(n=33)**			
	I am not aware of SA-DCP	15 (46)	
	I prefer physical appointments	13 (39)	
	I prefer phone calls	10 (30)	
	I am aware of SA-DCP, but I forgot to log in	9 (27)	
	I do not know how to use electronic services	7 (21)	
	I do not want to use electronic services	6 (18)	
	The use of electronic services is generally difficult	6 (18)	
	I do not receive personal help through electronic services	5 (15)	
	Other reasons	4 (12)	
	I am concerned about the data security and protection of electronic services	3 (9)	
	My sleep apnea treatment and CPAP therapy are balanced, so I do not need to contact health care professionals through any communication channel.	3 (9)	
	My sleep apnea treatment and CPAP therapy are balanced, so I do not need additional information through any communication channel.	1 (3)	
	I do not feel the need to log into SA-DCP as part of my CPAP therapy	1 (3)	

^a^CPAP: continuous positive airway pressure.

### Patients’ Prefamiliarization With CPAP Therapy Before the Initial Guiding Session

A larger proportion of SA-DCP users had familiarized themselves with prior information about CPAP therapy before the initial guiding session of CPAP therapy than SA-DCP nonusers (43/58, 74% vs 16/33, 49%; *P*=.02; Table 2). Among the 48 SA-DCP users who familiarized themselves with information about CPAP therapy beforehand, 29 (67%) performed it through the SA-DCP (Table 2). Most SA-DCP nonusers (6/16, 38%) said they had received the preliminary information from a spouse or a relative who had already used a CPAP device. The other sources of information for both groups were the internet (6/59, 10%), private health care providers (3/59, 5%), primary health care units (2/59, 3%), and the Duodecim medical information database (2/59, 3%). SA-DCP users also received information from occupational health care units (2/43, 5%) and the Facebook sleep apnea support group (2/43, 5%). Correspondingly, SA-DCP nonusers received information from specialized care units (2/16, 13%), research articles (1/16, 6%), and AEC (1/16, 6%).

The initial guidance sessions of CPAP therapy were carried out with small groups of patients (4-8 patients at a time). According to HCPs, the initial guiding sessions were smoother for patients who had already familiarized themselves with prior information about CPAP therapy through the SA-DCP (Textbox 1). The problem was that many patients still did not have prior information about CPAP therapy; therefore, most of the initial guiding sessions had to be implemented according to their needs. According to the HCPs, patients found the instructional videos available in the SA-DCP to be useful and clear (Textbox 1). Patients were also instructed to familiarize themselves with them and other information material found on the SA-DCP even after the sessions if they had further questions.

### Patients’ Information Needs About Sleep Apnea and CPAP Therapy

SA-DCP includes electronic messaging functionality between patients and HCPs and information about sleep apnea, its treatment, and CPAP therapy. According to the survey responses of SA-DCP users, most patients looked for information about the SA-DCP, sleep apnea, self-treatment of sleep apnea, and cleaning and maintenance of the CPAP device (Table 5). The messaging functionality of the SA-DCP and its “frequently asked questions” function were not widely used; only 38% (22/58) of SA-DCP users used them (Table 5).

**Table 5:** Functionalities of the sleep apnea digital care pathway (SA-DCP) used by patients according to the SA-DCP users survey (N=58).

		Values, n (%)
**The number of patients who familiarized themselves with the following information materials**		
	Welcome to SA-DCP	55 (95)
	Sleep apnea	45 (78)
	Cleaning and maintenance of the CPAP^a^ device	43 (74)
	Self-treatment of sleep apnea	42 (72)
	CPAP therapy	39 (67)
	Preparing for the CPAP therapy initial guiding session	38 (67)
	Sleep apnea and driving ability	32 (55)
	CPAP therapy in unusual everyday situations	28 (48)
	Controls, rehabilitation, and social security	25 (43)
	Frequently asked questions	22 (38)
The patient has communicated with the HCP^b^ in matters related to his or her treatment through the messaging functionality of SA-DCP		22 (38)

^a^CPAP: continuous positive airway pressure.

^b^HCP: health care professional.

During the treatment period, both SA-DCP users and SA-DCP nonusers sought additional information regarding their treatment without contacting HCPs (23/58, 39% vs 9/33, 27%; *P*=.48; Table 2). Among 23 SA-DCP users who sought more information, 8 (35%) performed it through the SA-DCP. The other reported information sources for SA-DCP users were the internet (9/23, 39%), Facebook sleep apnea support group (1/23, 4%), patient organizations (1/23, 4%), rehabilitation (1/23, 4%), and professional education (1/23, 4%). SA-DCP nonusers received additional information from the following sources: the internet (5/9, 56%), Facebook sleep apnea support group (1/9, 11%), scientific articles (1/9, 11%), and information material provided by private health care services providers (1/9, 11%). Most SA-DCP nonusers and SA-DCP users who sought more information about CPAP therapy and sleep apnea received the information they needed (8/9, 89% vs 21/23, 91%; *P*>.99; Table 2).

### Patients’ Communication Practices With HCPs

During the treatment period, 52% (17/33) of SA-DCP nonusers and 57% (33/58) of SA-DCP users contacted HCPs (Table 2). Among 33 SA-DCP users who contacted HCPs, 17 (52%) used the SA-DCP, and the rest used other contact methods (Table 2). SA-DCP users who preferred contact methods other than SA-DCP messages were older (mean 63.3, SD 9.4 vs mean 55.7, SD 10.8; *P*<.04). Phone calls were the most important form of contact for SA-DCP users (10/33, 30%). SA-DCP nonusers (9/17, 53%) mostly contacted HCP via phone calls. The next most common contact method for both groups was a physical visit to an AEC or a primary health care unit. The contact mostly concerned CPAP therapy; this was the case for 88% (15/17) of SA-DCP nonusers and 70% (23/33) of SA-DCP users (Table 2). Most SA-DCP nonusers and SA-DCP users who contacted HCPs received the help they needed (16/17, 94% vs 31/33, 94%; *P*=.41; Table 2).

According to the phone call register data, the annual number of phone calls to an AEC was still high even after the introduction of SA-DCP (Table 1). An exact comparison of phone calls to AECs per patient between different years could not be made because the number of new CPAP therapies in the OUH varied between different years, and the number of annual phone calls also showed contacts with HCPs from patients whose CPAP therapies had started in previous years (Table 1). The results of 2019 mainly represent a situation in which the SA-DCP was not yet in use at OUH because it was introduced at the very end of 2019. The results of 2021 represent a situation in which the SA-DCP had been in use at OUH for approximately 2 years. HCPs also indicated the same; there was no significant decrease in the number of phone calls, and there were still many phone calls related to CPAP therapy (Textbox 1). HCPs emphasized that they try to guide patients during phone calls to use the SA-DCP more in matters related to their care. Although the patients’ affairs were handled mostly with phone calls, the HCPs thought the instructional videos and informational materials included in the SA-DCP were valuable. It was possible to better explain things to patients with them (Textbox 1). For example, HCPs may instruct the patient during a phone call to watch SA-DCP's educational video to get a better understanding of the matter. The HCPs also emphasized that the SA-DCP is a good way to deliver the necessary contact information to patients, allowing them to reserve time for the necessary procedures themselves (Textbox 1).

### Patient Adherence to CPAP Therapy

According to the patients’ responses to the survey and remote monitoring data of CPAP devices, adherence to CPAP therapy was high in both groups (Tables 2 and 3). Both groups used the CPAP device on average for >6 hours per night and on >90% of nights (Tables 2 and 3). On the basis of the patients’ own assessments, adherence to CPAP therapy was high in both SA-DCP nonusers (mean 4.8, SD 0.5) and SA-DCP users (mean 4.7, SD 0.8; Table 2). In addition, according to the patients’ survey, 72% (42/58) of SA-DCP users reported that SA-DCP had made them more motivated to perform their own CPAP therapy (Table 2). Most patients in both groups believed that CPAP therapy helped them treat sleep apnea (Table 2). The remote monitoring data of CPAP devices showed that CPAP therapy had significantly reduced the number of AHIs for both groups (Table 3).

### Patient Feedback About SA-DCP

**Figure 1:**
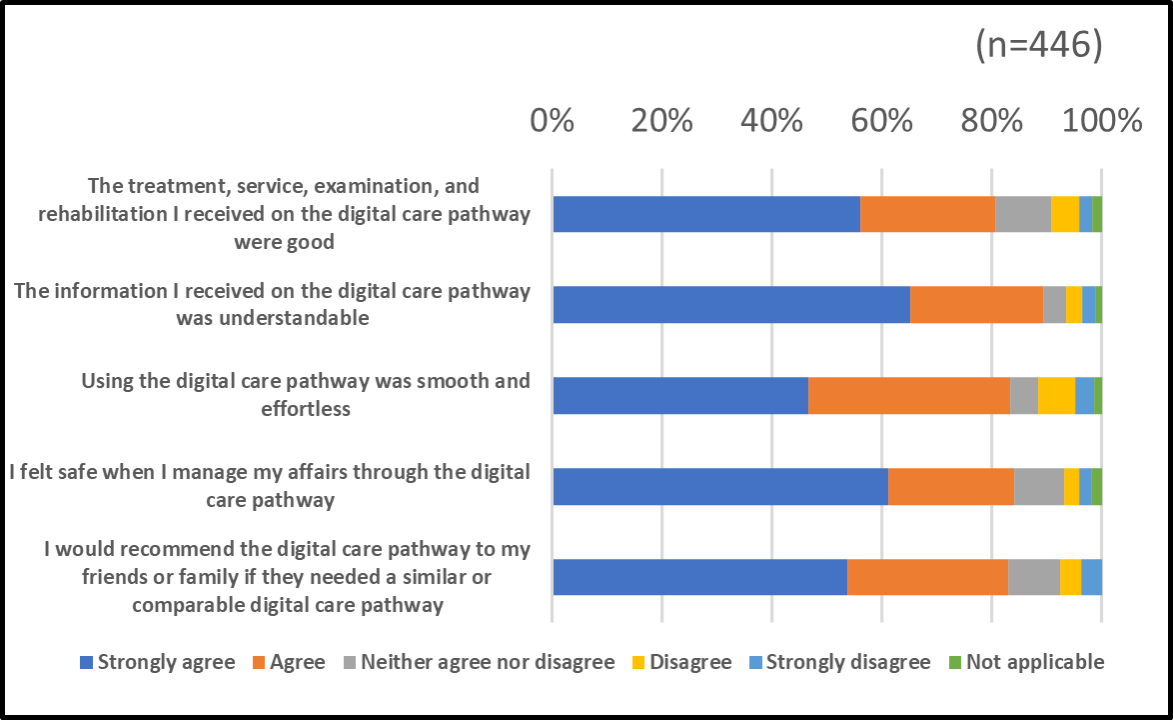
Patient feedback about the sleep apnea digital care pathway.

A total of 446 patients responded to the customer feedback survey; their feedback is shown in Figure 1. Patient feedback on the SA-DCP was generally positive; most of them agreed or strongly agreed with the survey claims (Figure 1). When examining the results, it should be noted that the questions of the patient feedback survey are common to every DCP in the OUH. As the functionalities offered by DCPs vary according to the care chains of different diseases, not all the questions are necessarily valid for every DCP. For example, examinations are not offered through the SA-DCP.

Of 446 patients, 102 (22.9%) who responded to the survey provided free-form feedback on the SA-DCP. Moreover, 22 patients gave generally positive feedback about the SA-DCP. For the most part, they did not elaborate on their feedback. According to 2 respondents, the possibility to use the services remotely was a good thing, and according to 2 respondents, the SA-DCP was a good and modern service. However, 19 patients thought that they did not need to use the SA-DCP, or that it did not add value to their treatment. Moreover, 11 respondents mentioned that communication and information about the SA-DCP should be improved. According to 9 respondents, the SA-DCP contained good and comprehensive information about sleep apnea and its treatment, as well as CPAP therapy. However, 3 respondents mentioned that although the SA-DCP contained good information, the same information can be found on the internet. With regard to SA-DCP’s messaging feature, 5 respondents thought it was a functional solution. Conversely, 9 respondents said that they encountered problems or delays related to messaging and 9 respondents desired new features for the SA-DCP, such as better search functionality. As the information content of SA-DCP was only available in Finnish during the research, some respondents presented English language support as a need for future development. According to 5 responses, SA-DCP’s user interface was clear, and its usability was good. In contrast, 4 respondents stated that the user interface could still be improved. Three respondents had technical problems and challenges when using the SA-DCP. Two users reported that the SA-DCP worked well technically. Three respondents said that they would not like to manage their affairs through digital services. Four respondents reported that they had experienced challenges using the SA-DCP, especially in relation to finding their own care path.

## Discussion

### Principal Findings

This study investigated whether the 3 main goals for introducing the SA-DCP at OUH were achieved. The first aim of introducing the SA-DCP was to shorten the initial guiding sessions of CPAP therapy on the assumption that the patients would have familiarized themselves with prior information about CPAP therapy in advance through the SA-DCP. The second main aim was to reduce the number of patients’ phone calls and contacts to HCPs, especially when the information can be found in the SA-DCP. The primary goal of implementing SA-DCP at OUH was to improve patients' adherence to CPAP therapy. However, according to the results of this study, not all the objectives of introducing the SA-DCP were achieved.

On the basis of the HCP’s responses to this study, shortening the initial guiding sessions of CPAP therapy had not been fully achieved, although a significantly larger number of SA-DCP users had familiarized themselves with prior information about CPAP therapy compared with SA-DCP nonusers. In this regard, it can be said that SA-DCP has contributed to the better preparation of patients for sessions. The initial guiding sessions were smoother for patients who had already familiarized themselves with prior information regarding CPAP therapy through the SA-DCP. However, many patients still did not have prior information about CPAP therapy; therefore, most sessions had to be implemented according to their needs. Because digital services may require care process changes to get the most out of them, 2 HCPs mentioned that the initial guidance sessions could also be carried out remotely in the future; however, this would require that the patients for whom this procedure would be suitable should be identified in advance [11,12].

Despite previous studies showing that DCPs would make it possible to reduce the number of patient phone calls to HCPs, this did not happen in the case of SA-DCP [37,38]. The annual number of phone calls to an AEC was still high even after the introduction of SA-DCP, according to the phone call register data. As the number of patients’ phone calls related to CPAP therapy was still high, HCPs mentioned that it was difficult to assess the actual change in the number of phone calls. However, they perceived that the number of patient calls did not decrease significantly. Previous studies have shown that patients’ ability to use electronic services also promotes the use of digital health care services [39,40]. However, Jenssen et al [41] found that despite the regular use of new digital technologies and services such as electronic banking, few of their study participants supported using these tools for communicating with their HCPs. The same behavior pattern can also be observed in the case of SA-DCP. Although SA-DCP users in this study perceived their ability to use ICT to be good and used computers, tablets, or smartphones regularly and were accustomed to using electronic services, only approximately half of them contacted the HCP with SA-DCP messages when needed. Among SA-DCP users, phone calls were the most important other contact method. The notable finding was that SA-DCP users who preferred another contact method were older.

Patient concerns about data security and protection have weakened their willingness to use electronic communication methods in health care [15,42]. On the basis of this study, this would not be an explanatory factor for the low use of SA-DCP messages, as both SA-DCP users and SA-DCP nonusers were not significantly concerned about the data security and protection of digital health care services. Zanaboni and Fagerlund [43] discovered that communicating via electronic tools was less time-consuming from the patient’s perspective than communicating via phone calls. However, some participants indicated that the time elapsed to receive a response from the HCP was more important than the time spent using the service itself. Long response times have been seen as one of the most important reasons for patients’ dissatisfaction with electronic communication in health care [39,44]. In a Norwegian study, older patients hoped that their electronic messages would be answered the next day at the latest; otherwise, they experienced dissatisfaction with the service [39]. From the patients’ point of view, they may perceive that a phone call is a quick and convenient way to handle their health-related matters [45-47]. The fundamental difference is that a phone call involves real-time interactive communication, whereas SA-DCP messages can be defined as asynchronous communication [48]. The patient may ask follow-up questions during the phone call and the HCP can answer them immediately. When using electronic communication tools, there may be delays in answers to questions and possible follow-up questions because of asynchronous communication, as the patient and the HCP may not be dealing with the issue simultaneously [48].

One of the main goals of introducing the SA-DCP was to improve patients’ adherence to CPAP therapy. This study showed no statistical difference between SA-DCP users’ and nonusers’ adherence to CPAP therapy. Adherence to CPAP therapy was high in both groups according to the patients’ own estimates and remote monitoring data of CPAP devices. Both groups performed CPAP therapy regularly and reported that it helped them to treat their sleep apnea. In addition, 72% (42/58) of SA-DCP users reported that SA-DCP motivated them to perform their own CPAP therapy. Unfortunately, this study did not ask why the participants felt this. The role of the SA-DCP was to complement CPAP therapy by providing information and an electronic communication channel. It did not include clear mechanisms for influencing patients’ behavior related to their own health as digital health interventions typically do, for example, in relation to weight management [49-51]. The CPAP therapy clearly helped the participants in this study to reduce the number of AHIs. Presumably, the biggest motivation for performing CPAP therapy came from alleviating sleep apnea symptoms and not so much from using the SA-DCP; therefore, the SA-DCP was not a significant factor in explaining adherence to CPAP therapy.

This study investigated HCPs’ perspectives on the SA-DCP and its usefulness for their work. Although previous studies determined that DCPs could potentially free health care services capacity for other purposes and reduce the workload of HCPs, the results of this study do not support these results in the case of SA-DCP [27,28]. The HCPs who participated in the study were unable to define significant changes in their workload and work practices after the introduction of SA-DCP. The primary aim of HCPs was for patients to use the SA-DCP more so that its benefits could be better used. Previous studies have highlighted that DCPs can promote work flexibility, for example, by enabling HCPs to respond to patients’ DCP messages at nonurgent, not only prereserved times [27,31]. The responses of HCPs in this study pointed out the same. With the help of the DCP, patients can access the information it contains before and after contact with HCPs, thus reducing patient follow-up questions [12]. From this perspective, HCPs felt that educational videos and information materials on SA-DCP were beneficial because, through them, the patients could better understand things. From a technical point of view, the SA-DCP’s weak integration with existing ICT systems was seen as one of its key shortcomings and an area for future development. The lack of interoperability with existing ICT systems has been found to weaken the willingness of HCPs to use digital health care services and increase their workload [10,52]. According to the interviewed HCPs, lack of integration reduced the fluency of their work, increased the workload of one responder, and can cause risks from the perspective of information protection and patient safety when patient information is copied manually between different programs.

Digital health care services are intended to help patients become more active actors, more adherent to their own care, and change their behavior in a more favorable direction for their health [49-51,53,54]. Promising results have already been achieved, for example, in treating obesity with the help of digital services [30,51]. In the case of SA-DCP, it was hoped that patients would be active and familiarize themselves with the information contained in it about sleep apnea and CPAP therapy. According to the patient survey, most SA-DCP users have done so. Although most SA-DCP users familiarized themselves with the information in SA-DCP, there was no statistically significant difference in the proportion of SA-DCP users and nonusers who sought additional information about their illness or CPAP therapy. From this perspective, it cannot be said that SA-DCP users are more active actors. It has been established that digital health care services can lower the threshold for patients to contact HCPs [37,54,55]. According to this study, there was no statistically significant difference between the percentage of SA-DCP users and SA-DCP nonusers who contacted HCPs during their treatment period. However, this study did not ask how often the patients contacted the HCPs. On the basis of the results of this study, it seems that patients sought additional information about their illness or contacted HCPs when they had a real need, regardless of the information source or communication method.

Most SA-DCP users thought that the treatment they received through the SA-DCP was good; it was fine technically, a safe service, and the information it contained was clear and understandable. However, some patients still did not use SA-DCP, although the relative number of active SA-DCP users increased during the study period. Lack of digital literacy is one of the barriers to promoting the use of digital health care services. Older adults, in particular, tend to have lower digital literacy than the general population [39,56]. Mannheim et al [40] emphasized in their study that older adults are not a homogeneous group in terms of digital literacy and should also be better included when designing digital health care services [40]. On the basis of the patient survey, there was no statistically significant difference in the age of SA-DCP users and SA-DCP nonusers, but based on remote monitoring data from CPAP devices, SA-DCP nonusers were older. According to this study, SA-DCP nonusers perceived their abilities to use ICT to be worse; they used computers, tablets, or smartphones more rarely and were less accustomed to using electronic services than SA-DCP users. SA-DCP nonusers preferred phone calls or physical appointments to manage their health-related issues with HCPs. The results showed a statistically significant difference in how clearly the patients perceived the communication about SA-DCP. Only 24% (7/38) of SA-DCP nonusers considered communication to be clear, and ignorance of the SA-DCP was the most common reason for them not to use the SA-DCP. After the diagnosis of sleep apnea, the patients received an information letter containing information about the disease and its treatment. This letter also included information on the SA-DCP and how to use it. Did SA-DCP nonusers think the SA-DCP was not adequately explained because they did not want to use digital health care services in the first place and preferred to conduct their health-related issues through phone calls or physical visits? They may not have paid attention to the SA-DCP information letter if they do not typically use or are not willing to use digital health care services or if they perceive they have weak skills in using them.

One of the key findings of this study is that the nonuse of SA-DCP and its functionalities among patients with sleep apnea means that its full potential is not being used. This can be seen, for example, in the initial guiding sessions of CPAP therapy, when some patients still come without prior knowledge. Although the number of SA-DCP users increased during the years covered by this study, not all SA-DCP functionalities were significantly used. In particular, this was reflected in the fact that SA-DCP messages were not widely used; therefore, the number of calls to AECs was not reduced. This study found that lower digital literacy and older age were significant factors in explaining the nonuse of the SA-DCP. Older SA-DCP users more often favored other contact methods, such as phone calls, when contacting HCPs during their treatment period. In the future, special attention should be paid to how digital health care services are designed according to the needs of older adults with weak digital literacy. Care processes should be better adapted to the requirements of digital health care services. Clearly, only the traditional information letter about SA-DCP is not sufficient to encourage all patients to adopt it. If there are challenges in deployment, patients could be more actively encouraged to adopt the SA-DCP and offered support. Previous studies have highlighted that the desire of older adults to use digital health care services can be supported by offering guidance and peer support [39,56]. Studies have also emphasized that both professionals and patients should be closely involved in DCP development to obtain the best benefit and that development should be a continuous process [10,29]. With age, various functional limitations, such as diminished eyesight related to diabetes or deteriorated motor skills owing to rheumatism, can increase and thus make it more difficult to use digital services [57,58]. Therefore, special attention should be paid to the usability and accessibility of digital services. The real end users should be involved in the design process, as the interviewed HCPs highlighted [22,25,58].

### Limitations

Our study had some limitations. Patients with sleep apnea give up CPAP therapy for different reasons, which can bias this study’s data regarding patients’ adherence to CPAP therapy. Most patients who responded to the survey had continued CPAP therapy for ≥1 year, and remote monitoring data on CPAP devices were collected in connection with 1-year control. Unfortunately, when the study was carried out, no information was available on the proportion of SA-DCP users and SA-DCP nonusers who had discontinued CPAP therapy. This would have provided additional information about patients’ adherence to CPAP therapy. Previous results have highlighted that high attrition rates hinder achieving the full benefits of digital health care services. During the implementation of the study, the SA-DCP did not enable the automatic collection of log data on the activity of patients using the SA-DCP, but through the automatic log data, it was only possible to determine that the patient had used the SA-DCP. Therefore, this study did not examine patients’ adherence to SA-DCP use, but only whether they had used the service.

On the basis of the study’s results, approximately half of SA-DCP users still contacted HCPs in a way other than through SA-DCP messages, although they reported having good digital literacy. Most SA-DCP nonusers also preferred phone calls to contact HCPs. However, in this study, SA-DCP users and SA-DCP nonusers were not asked why some preferred phone calls to contact HCPs instead of electronic messaging. Future research is needed to better understand this behavior pattern. This study did not ask patients how many times they contacted HCPs; it only investigated whether the patients contacted HCPs during their treatment period. Information on the number of contacts would have provided valuable information on whether using the SA-DCP can lower the threshold for contacting HCPs.

One of the goals of the SA-DCP was to increase patients’ adherence to CPAP therapy, and most SA-DCP users felt this was the case. Although the results of the survey and the remote monitoring data of the CPAP devices showed that there was no statistically significant difference in adherence to CPAP therapy between the groups, it would have been beneficial to ask SA-DCP users why most of them felt that the SA-DCP had increased their adherence to CPAP therapy. However, this was not investigated in this study. The sample size of the interviewed HCPs was small in this study. However, the answers to the HCPs were mostly consistent. Most of them thought there were no significant changes to their workload and work practices; there were still many phone calls from patients. At the time of writing, the SA-DCP did not enable the automatic collection of log data about the number of electronic messages. If this information had been available, it would have enabled a better comparison between the volumes of phone calls and SA-DCP messages.

### Conclusions

According to this study, not all the goals set for introducing the SA-DCP have been achieved. The HCPs who participated in the study could not define significant changes in their workload and work practices after the introduction of SA-DCP. The SA-DCP has brought more flexibility to HCPs’ work practices regarding patient communication. Despite using SA-DPC, some patients still wanted to communicate with HCPs by phone. Adherence to CPAP therapy was high in both SA-DCP users and nonusers. Patients’ lower digital literacy and older age were the most significant factors explaining the nonuse of the SA-DCP. In the future, more attention should be paid to how these user groups should be considered in the design and introduction of the DCPs.

## Abbreviations

AEC: assistive equipment center
AHI: apnea-hypopnea index
CPAP: continuous positive airway pressure
DCP: digital care pathway
HCP: health care professional
ICT: information and communications technology
OUH: Oulu University Hospital
SA-DCP: sleep apnea digital care pathway
